# 6-Shogaol attenuated ethylene glycol and aluminium chloride induced urolithiasis and renal injuries in rodents

**DOI:** 10.1016/j.sjbs.2021.03.005

**Published:** 2021-03-14

**Authors:** Muhammad Afzal, Imran Kazmi, Anwarulabedin Mohsin Quazi, Aftab Ahmad, Fahad A. Al-Abaasi, Faisal Imam, Khalid Saad Alharbi, Sami I. Alzarea, Ameeduzzafar Zafar

**Affiliations:** a Department of Pharmacology, College of Pharmacy, Jouf University, Aljouf 72341, Saudi Arabia; b Department of Biochemistry, Faculty of Science, King Abdulaziz University, Jeddah 21589, Saudi Arabia; c Health Information Technology Department, Faculty of Applied Studies, King Abdulaziz University, Jeddah 21589, Saudi Arabia; d Department of Pharmacology and Toxicology, College of Pharmacy, King Saud University, Riyadh, Saudi Arabia; e Department of Pharmaceutics, College of Pharmacy, Jouf University, Aljouf 72341, Saudi Arabia; f Glocal School of Pharmacy, Glocal University, Saharanpur, Uttar Pradesh, 247121, India

**Keywords:** 6-Shogaol, Calcium oxalate, Ethylene glycol, Urolithiasis

## Abstract

The 6-shogaol, is a flavanone type flavonoid that is abundant in citrus fruit and has a wide range of pharmacological effects. The present study attempted to evaluate the antiurolithic effect of 6-shogaol on ethylene glycol (EG) and ammonium chloride (AC)-induced experimental urolithiasis in rats. The efficacy of 6-shogaol 50 mg/kg and 100 mg/kg was studied in EG 0.75% (V/V) and AC 1% (W/V) experimentally induced urolithiasis in rats for 21 days. The weight difference, urine volume, the levels of calcium, phosphate, magnesium, oxalate and uric acid in urine was observed. The blood urea nitrogen, creatinine, uric acid in serum and levels of malondialdehyde (MDA) and glutathione (GSH) were also measured. Histopathological analyses in kidneys were also performed. The rats weights were higher in the 6-shogaol groups than the urolithiasis group. EG caused a significant increase in serum creatinine (p < 0.05), BUN (P < 0.001), and uric acid (p < 0.01) while treatment with Cystone (750 mg/kg), and 6-shogaol (50 and 100 mg/kg) showed the significant reduction in increased serum levels of creatinine (p < 0.001), uric acid (p < 0.01) and BUN (p < 0.001). Administration of EG and AC showed statistically significant (p < 0.001) elevated levels of MDA and reduction in GSH levels. Treatment of Cystone (750 mg/kg), and 6-shogaol (50 and 100 mg/kg) significantly (p < 0.001) reduced MDA levels and an increase GSH levels as compared to EG and AC-treated group. The histological findings further attested antiurolithiatic properties of 6-shogaol. The present study attributed clinical shreds of evidence first time that claiming the significant antiurolithic effect of 6-shogaol and could be a cost-effective candidate for the prevention and treatment of urolithiasis.

## 1 Introduction

The occurrence of calculus in the kidney and other sections of the urinary tract, mainly the bladder and ureter are known as urolithiasis. Calculi that formed in the urinary tract in the majority (80%) of the cases is composed of phosphate and oxalate crystals of calcium (Tiselius, 2003). The previous reported studies also postulated that the rate of recurrence of urolithiasis is about 10% per year in cases with the least preventive and precautious measures (Basavaraj et al., 2007). Epidemiology study postulated that the rate of prevalence of urolithiasis in males (8–19%) is more than females (3–5%) in Western countries (Trinchieri, 2008). A previously reported study identified various pathogenic processes that are involved in urolithiasis such as aggregation, crystal nucleation, and formation of insoluble particles (Baumann, 1998).

Clinically observed that excessive accumulation of insoluble saturated particles as a consequence of the high rate of urinary execration tends to the occurrence of kidney stones (Evan, 2010). The rate of incidences of urolithiasis has been drastic increases globally over the period, as per the previously recorded data it some countries as follows in Italy 2.4%, China 2.5%, USA 3.6%, India 4%, UK 4.5, Taiwan 5% and in Turkey with highest rate i.e. 14.8%, (Alatab et al., 2016) with remarkably 50 of cases showing the relapses (Bouatia et al., 2015).

The primary idiopathic risk factor for the occurrence of urinary stones is hyperoxaluria whereas crystal of calcium oxalate is one of the major identified constituents for the pathology of kidney stones (Neisius and Preminger, 2013). The major identified primary risk factor for kidney stones is hyperoxaluria, whereas CaOx is considered one of the major constituting elements of urinary stones (Neisius and Preminger, 2013). Increased levels of CaOx crystals resulted in injuries to the renal tubular cells because of abnormal formation of ROS and remarkably development oxidative stress (Zhang et al., 2017). The molecular mechanism involved in generating reactive oxygen species is carried forward by the enzymatic process mainly through NADPH i.e. nicotinamide adenine dinucleotide phosphate oxidase intracellularly through various signaling pathways. A principal origin of receptor-linked reactive oxygen species production is NADPH oxidase in the presence of high levels of CaOx crystals (Khan, 2013, Sedeek et al., 2013).

In an attempt to prevent relapses and recurrence of urinary stones, very less significant efficacy regimens are commercially available, including thiazide diuretics and alkali-citrate. The plant products listed in the traditional systems of medicine especially Ayurveda, promisingly decrease the rate of recurrences of renal calculi (KVSRG et al., 2007).

A well-known traditional medicine, rhizomes of ginger have been used to deal with numerous ailments. The rhizomes of Zingiber *officinale* known to exert various pharmacological activities such as neuroprotective and anti-neuroinflammatory as it contains one of the important bioactive pungent compound 6 shogaol (Seow et al., 2017). The bioactive principles of gingerols (4-,6-,8,10-, and 12-gingerols) and the shogaols (including 6-, 8-, and 10-shogaols) are important identified constituents of which are known to have a variety of pharmacological actions (Semwal et al., 2015),(Kou et al., 2018). Recent research reports suggest the therapeutic values of 6-Shogaol as anticancer (Yi et al., 2019), potent anti-inflammatory and antioxidant (Dugasani et al., 2010), antidiabetic (Yi et al., 2019), and antiemetic (Kou et al., 2018).

## 2 Methodology

### 2.1 Animals

As per the prescribed guidelines for the care and handling of animals, the male Wistar rats (180–220 g) were housed and kept at 12/12 light: dark cycle mode under prescribed laboratory environments in specialized cages made up of polypropylene. During the experimental protocol, tap water with a normal pelleted diet was used to feed the animal's ad *libitum*. Approval for the conduction of experimental protocol was obtained (RKDFCP/IAEC/2020/33) from the Local committee of bioethics and all experiments conducted according to the guidelines of Committee for the purpose of control and safety on experimental animals (CPCSEA), India. All experiments carried out at RKDFCP, India.

### 2.2 Induction of urolithiasis

Assessment of antiurolithiatic activity in urolithiasis animal model induced by EG and AC concurrently in male albino Wistar rats as per earlier reported methods with slight modifications. In 21 days protocol, 0.75% (V/V) & 1% (W/V) ethylene glycol together with ammonium chloride supplied through drinking water ad libitum for induction of urolithiasis associated with the formation of crystal of calcium oxalate (Bano et al., 2018).

### 2.3 Experimental design

In a present experimental study, six animals in each group of a total of five groups were selected and maintained on standard pallet fed. The experimental groups for assessment are categorized into, normal treated groups, inducer group, standard treated, and test group respectively. In Group I, animals were supplemented tap water with pellet fed for 21 days. The Group II animals have received a dose of 0.75% for EG V/V simultaneously 1% of ammonium chloride W/V in tap water with pellet fed for 21 days. The Group – III animals were given a dose of 0.75% for EG V/V simultaneously 1% of ammonium chloride in water and along with standard drug Cystone with a dose of 750 mg/kg, through gastric gavage orally for 21 days at the interval of 24hrs. Similarly, in 6-shogaol treatment groups (Groups IV, and V); animals were exposed to ethylene glycol (EG) 0.75% V/V and ammonium chloride (AC) 1% W/V in water and 50, and 100 mg/kg doses of the 6-shogaol through gastric gavage orally for 21 days at the interval of 24hrs, along with weekly monitoring and recording of body weight of all animals. The metabolic cages were employed for the collection of 24hrs urine samples from experimental animals on the 21st day of assessment. Collection of blood through the *retro*-orbital sinus of rats was carried out for the serum analysis. As per previously reported data after the completion of experimental protocols, the animals were sacrificed both the kidneys were dissected, excised, and stored in formalin and subject to further histopathological investigations.

### 2.4 Assessment of antiurolithiatic activity

#### 2.4.1 Body weight

Bodyweight was recorded for each rat by every week, the recorded body weight expressed in terms of RBW, i.e. relative body weight. The formula for computing RBW efficiently required two parameters to get assess which include IBW initial body weight and ABW absolute body weight respectively (Bouanani et al., 2010).

ABW (g)
RBW =  — × 100
IBW (g)

#### 2.4.2 Urine analysis

The metabolic cages were employed individually for the collection of 24 hrs urine samples from all experimental animals on the 21st day of assessment. During the period, rats had free drinking water access. After collection of urine samples in 24 hrs were subjected under a 10X microscope for examination of crystals morphology. The total urine volume, calcium, phosphate, magnesium, oxalate, and uric acid were analyzed.

#### 2.4.3 Serum analysis

Post 21 days experimental procedure, animals were anesthetized and blood was collected from the *retro*-orbital sinus and subjected to centrifuge for 10 min. at 3000 rpm. Serum obtained was stored at −20 °C until further biochemical parameters estimation such as creatinine, blood urea nitrogen (BUN), and uric acid by using different biochemical estimation kits.

#### 2.4.4 Kidney homogenate analysis

The kidneys of rats were excised and isolated. They were washed off superfluous tissue and swilled in physiological saline ice-cold and 50% portion of the isolated kidney was kept in crushed ice. They were crosscut into fine slices with a surgical scalpel and were chilled with cold 0.25 M sucrose, easily blotted with filter paper. The homogenate tissues 10% (w/v) was prepared in 0.1 M Tris hydrochloride buffer (pH 7.4) in a homogenizer at a speed of 2500 rpm. The homogenate was centrifuged for 20 min (−4 °C) at 5000 rpm using a cooling centrifuge. The clear supernatant was obtained after centrifuged and used to estimate Malondialdehyde (MDA) and glutathione (GSH) (Lulat et al., 2016).

#### 2.4.5 Histopathology

The histological examination was carried out by preparing different sections of the kidney after each sacrifice; the kidney specimens was taken from all groups and washed with saline, and fixed in phosphate-buffered formalin solution. The specimen was embedded in paraffin and subjected to thick sections followed by staining with eosin and hematoxylin. The specimen was viewed under the polarized light microscope for histopathological examination.

#### 2.4.6 Statistical analysis

The readings from study protocols were subjected to analysis by parametric tests using one-way variance analysis with post hoc Tukey's test. The values obtained were expressed in terms of mean ± standard error mean. A value of p < 0.05 was taken significantly on statistical parameters.

## 3 Results

### 3.1 Body weight

*Effect of 6-shogaol on RBW in EG and AC-induced urolithiasis in rats*

One-way variance analysis with post hoc Tukey's test discloses that except the EG and AC-treated group other test group rats exerted significant increase in relative body weight (RBW). The EC and AC- treated group explores a remarkable decrease p < 0.01 in relative body weight in rats. Cystone standard control and the 6-shogaol (100 mg/kg/BW) treated group (Table 1) significantly prevented this detrimental disease progression.

**Table 1:** Effect of 6-shogaol on relative body weight in ethylene glycol and ammonium chloride-induced urolithiasis rats.

**Relative Body Weight (%)**	**Group-I (Normal)**	**Group-II Control (EG and AC-1% w/v)**	**Group-III (Cystone treated 750 mg/kg)**	**Group-IV (Test-I) (6-shogaol 50 mg/kg)**	**Group-V (Test-II) (6-shogaol 100 mg/kg)**
**Week 1**	115.1 ± 3.5	100.3 ± 2.5	100.3 ± 2.5	100.5 ± 6.5	109.2 ± 2.8
**Week 2**	122.2 ± 4.5	100.1 ± 5.5^#a^	120.2 ± 4.0^**b^	116.3 ± 1.9^**b^	122.2 ± 4.5^**b^
**Week 3**	148.2 ± 6.5	108.4 ± 3.9^##a^	143.2 ± 5.5^***b^	130.3 ± 2.5^**b^	140.7 ± 4.1^***b^

Values given are ±SEM for (n = 6), ^#^p < 0.05, ^##^p < 0.01, ^###^p < 0.001vs. week 1 of respective groups; *p < 0.05, **p < 0.01, ***p < 0.001 ^a^vs Normal group, ^b^vs as compared to ethylene glycol (EG) and ammonium chloride (AC) control group.

### 3.2 Urine analysis

*Effect of 6-shogaol on urine parameters in EG and AC-induced urolithiasis in rats*

One-way variance analysis with post hoc Tukey's test postulated that treatment with standard drug Cystone (750 mg/kg) and, test drug 6-shogaol (50 or 100 mg/kg), on statistical correlation found to be significantly decrease p < 0.001 parameters such as water intake, the total volume of urine, and pH of urine. Moreover, urolithiasis pathology was associated with remarkably increased levels of urinary parameters such as uric acid (p < 0.001), magnesium (p < 0.001), calcium (p < 0.0001), and phosphate (p < 0.0001) concentration in urine of EG and AC treated groups. Whereas dose of Cystone (750 mg/kg), and 6-shogaol (50 and 100 mg/kg) showed a significant antiurolithic property by remarkably decreasing the levels of the above-mentioned increased urinary profile (Table 2).

**Table 2:** Effect of 6-shogol on urinary parameters in ethylene glycol and ammonium chloride-induced urolithiasis rats.

**Parameters (Units)**	**Group-I (Normal)**	**Group-II Control (EG and AC-1% w/v)**	**Group-III (Cystone treated 750 mg/kg)**	**Group-IV (Test-I) (6-shogaol 50 mg/kg)**	**Group-V (Test-II) (6-shogaol 100 mg/kg)**
**URINE VOLUME (ml/24 hrs)**	1.82 ± 0.15	5.70 ± 0.59^#^	2.37 ± 0.43^***^	3.15 ± 0.43^**^	2.87 ± 0.45^**^
**WATER INTAKE (ml/24 hrs)**	5.54 ± 0.50	26.81 ± 1.06^#^	6.04 ± 0.77^***^	11.50 ± 0.95^**^	7.71 ± 1.19^***^
**pH**	6.52 ± 0.03	9.88 ± 0.22^###^	6.88 ± 0.16^***^	7.39 ± 0.18*	6.95 ± 0.20^***^
**MAGNESIUM**	4.88 ± 0.03	1.91 ± 0.20^###^	3.70 ± 0.15^***^	2.93 ± 0.10	3.85 ± 0.29^***^
**URIC ACID (mg/24 hrs)**	2.75 ± 0.06	6.83 ± 0.12^###^	2.84 ± 0.03^***^	5.38 ± 0.49*	3.61 ± 0.26^**^
**CALCIUM (mg/24 hrs)**	1.32 ± 0.04	5.50 ± 0.03^###^	1.49 ± 0.02^***^	3.06 ± 0.39^**^	2.51 ± 0.24^***^
**PHOSPHATE (mg/24 hrs)**	5.64 ± 0.11	9.24 ± 0.19^###^	5.80 ± 0.06^***^	7.71 ± 0.35*	5.99 ± 0.17^***^
**OXALATE (mg/24 hrs)**	0.27 ± 0.01	2.20 ± 0.17^#^	0.54 ± 0.02^***^	1.43 ± 0.21*	1.02 ± 0.14^***^

Values given are ±SEM for (n = 6), one-way ANOVA followed by the Tukey test ^###^ p < 0.001 as compared to normal group, *p < 0.05, ^**^p < 0.01, ^***^p < 0.001.as compared to ethylene glycol (EG) and ammonium chloride (AC) control group.

### 3.3 Serum analysis

*Effect of 6-shogaol on serum parameters in EG and AC-induced urolithiasis in rats*

One-way variance analysis with post hoc Tukey's test also postulated that significant, high levels of creatinine (p < 0.05), BUN (P < 0.001), and uric acid (p < 0.01) were reflected in serum of EG treated group. Dose of Cystone (750 mg/kg), and 6-shogaol (50 and 100 mg/kg) showed the significant reduction in increased serum levels of creatinine (p < 0.001), uric acid (p < 0.01) and BUN (p < 0.001). Furthermore, EG and AC-treated group showed statistically significant (p < 0.001) elevated levels of MDA and reduction in GSH levels. However, Cystone (750 mg/kg), and 6-shogaol (50 and 100 mg/kg) treatment showed a significant (p < 0.001) reduction in MDA levels and an increase GSH levels as compared to EG and AC-treated group (Table 3).

**Table 3:** Effect of 6-shogol on serum blood parameters in ethylene glycol and ammonium chloride-induced urolithiasis rats.

**Parameters (Units)**	**Group-I (Normal)**	**Group-II Control (EG and AC-1% w/v)**	**Group-III (Cystone treated 750 mg/kg)**	**Group-IV (Test-I) (6-shogaol 50 mg/kg)**	**Group-V (Test-II) (6-shogaol 100 mg/kg)**
**CREATININE (mg/dl)**	0.82 ± 0.01	1.40 ± 0.02^##^	0.90 ± 0.06^***^	1.00 ± 0.01*	0.95 ± 0.01^***^
**BLOOD UREA NITROGEN (mg/dl)**	34.8 ± 1.27	60.1 ± 0.53^###^	36.8 ± 1.05^***^	40.93 ± 0.60^**^	38.75 ± 0.49^***^
**URIC ACID (mg/dl)**	1.85 ± 0.02	3.32 ± 0.06^#^	2.34 ± 0.03^***^	3.88 ± 0.49*	3.513 ± 0.36^**^
**Malondialdehyde (nmol/mg of protein)**	0.58 ± 0.12	4.78 ± 0.12^####^	1.01 ± 0.13^***^	3.5 ± 0.13*	2.5 ± 0.12^**^
**Glutathione(nmol/mg of protein)**	8.30 ± 0.90	5.50 ± 1.05^###^	8.01 ± 1.00^***^	6.50 ± 0.80^**^	7.30 ± 0.80^***^

Values given are ±SEM for (n = 6), one-way ANOVA followed by the Tukey test ^###^ p < 0.001 compared to normal *p < 0.05, ^**^p < 0.01, ^***^p < 0.001. as compared to ethylene glycol (EG) and ammonium chloride (AC) control group.

### 3.4 Histopathology

**Fig. 1:**
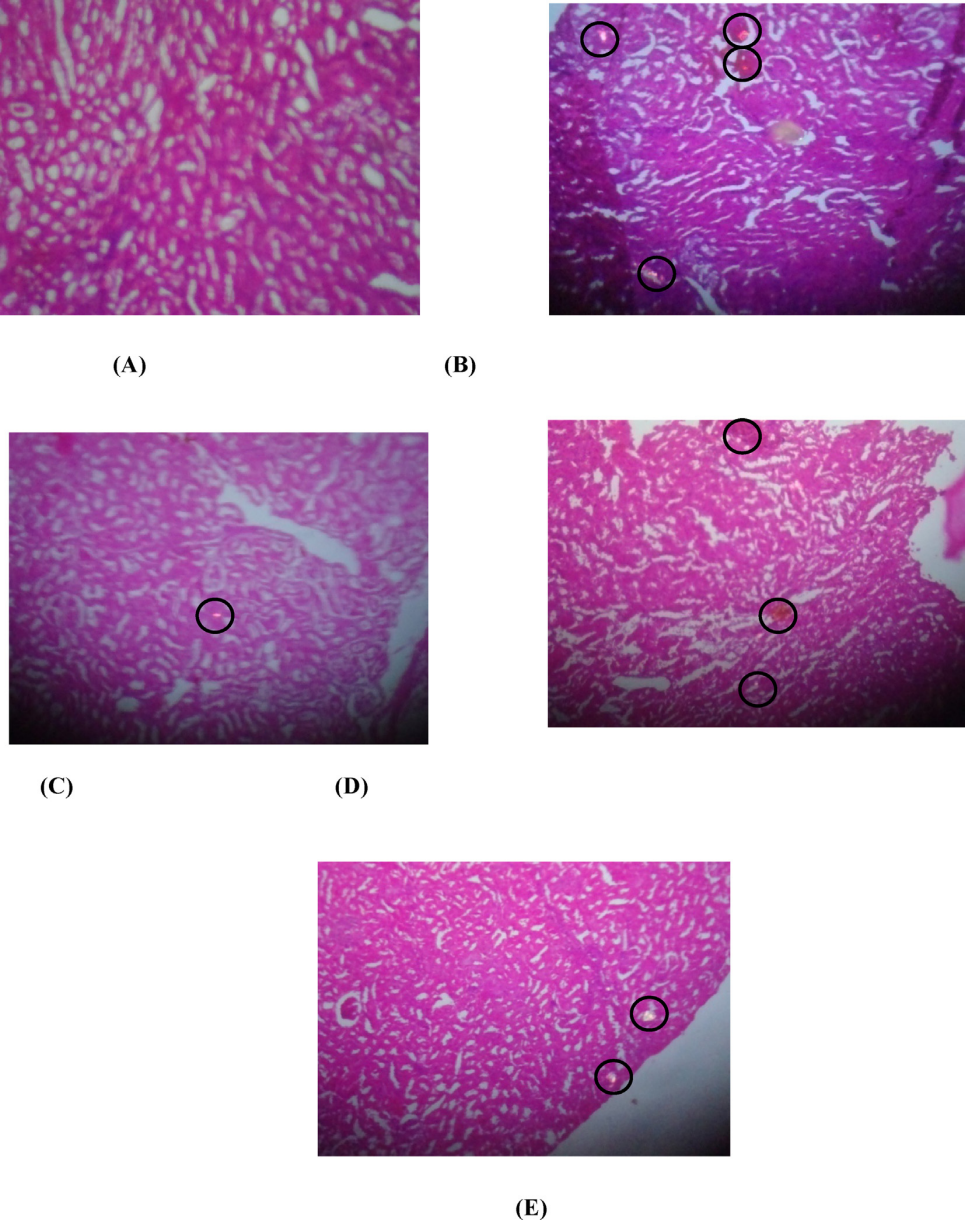
**Kidney Histopathology**: **A)** Normal Control group, **B)** Ethylene Glycol control group, **C)** Cystone 750 mg/kg treated group, **D)** 6-shogaol 50 mg/kg, **E)** 6-shogaol 100 mg/kg treated group, Circular sections indicate CaOx crystal depositions in respective sections.

Histopathologic examinations of renal tissue homogenate from EG treated group discloses deposition of crystal inside tubules of nephrons with remarkable deteriorating changes in both kidneys (Fig. 1 B). Cystone (750 mg/kg), and 6-shogaol (50 and 100 mg/kg) significantly attenuated elevated deposition of oxalate, phosphate, and calcium in renal tissue and ameliorated renal cellularity (Fig. 1 C, D, and E).

## 4 Discussion

The super saturation of urine with respect to stone formation substances is important clinical contributing factors in urolithiasis. EG-induced urolithiasis model has been generally used as an experimental and can be moderately inconsistent. However, AC-induced rats lead to metabolic acidosis. Hence, a high rate of CaOx deposition in the kidney with a combination of 0.75% of ethylene glycol (EG; V/V) and 1% ammonium chloride (AC; W/V) has been used in this study. The objective of the study to investigate the antiurolithic properties of 6-shogaol on EG and AC-induced experimental in rats.

The previously reported studies postulated that the length of stone deposition in female rats is lesser as compared to male rats in EG induced animal model(Prasad et al., 1993, Karadi et al., 2006). Hence, the present study extensively uses male rats. In the present study, 6-shogaol treated group significantly improve kidney function by imparting various urinary parameters as compared to EG and AC-treated groups. Given observations, the EG and AC-treated group showed remarkable elevation of urine volume, water intake, and pH. In another experimental animal group, it was observed that pre-exposure of Cystone and 6-shogaol are significant decreases elevated levels of urine, water intake, and pH in EG and AC-treated groups. The presence of urinary oxalate crystals resulted in increased urine volume through obstruction in urine flow as a result of this biological waste such as uric acid, creatinine, and BUN, which important biomarker for kidney damage. Previously reported studies also demonstrated that elevated levels of oxalate in urine has been responsible for lipid peroxidation and its interaction with polyunsaturated fatty acids in the cell membrane as one of the important identified cause for renal tissue damage (Selvam et al., 2001, Huang et al., 2002, Anand et al., 1994).

In the present study, EG and AC-treated group significantly increase intracellular calcium, phosphate, and 6-shogaol treated group was shown to decrease intracellular calcium. This clinical hypothesis may be due to increasing levels of nitric oxide intracellularly resulted in activation of enzyme cyclic monophosphate that is responsible for controlling intracellular calcium levels. In the present study, we estimated the effect of the EG and AC- treated group on intracellular levels of magnesium, which revealed that EG and AC-treatment significantly decreases the levels of magnesium as compared to the normal group. Whereas, in another set of the group it was postulated that Cystone and a higher dose of 6-shogaol 100 mg/kg significantly restore the intracellular magnesium levels. EC and AC-induced urolithiasis animal model showed the presence of irregular polymorphic oxalate crystals deposition inside the microtubules when kidney sections subjected to microscopic examinations. The irregular crystal may be the contributing factor for the inflammation of the interstitium and dilation of PCT (proximal convoluted tubules).

Moreover, EG and AC-treated group showed a significant increase in MDA levels and decreased GSH levels in our study. An earlier study reported that oxalate concentration increased in urine due to inducing lipid peroxidation and caused a glomerular as well as tubular damage(Lulat et al., 2016). However, treatment of Cystone and 6-shogaol prevented the reduction of GSH and elevation in MDA levels. This activity could be contributed by 6-shogaol due to the presence of an electrophilic α, β-unsaturated carbonyl moiety, which may show the antiurolithiatic activity against EG and AC-treated rats (Chen et al., 2014).

Histopathological findings of the present study postulated that treatment with 6-shogaol remarkably lowers the number and size of CaOx crystals in different microscopic structures of kidneys and significantly restores the normal kidney functions by preventing further damage to the kidneys. Among various other types of urolithiasis, the CaOx type is identified as the most prevalent and constantly growing type (Knoll et al., 2011). The previously reported study of *Daucus carota* in experimentally EG and NH4Cl-induced hyperoxaluria animal model, CaOx urolithiasis was addressed in this study (Albayrak et al., 2013, Tugcu et al., 2008). A well-known commercially available polyherbal formulation Cystone was used for the treatment of kidney ailments and as a standard control for the present study (Rao and Rao, 1998). The molecular mechanism involved in the antiurolithic effect of Cystone includes diuretic, smooth muscle relaxant, and its clinical ability to dissolve the polymorphic crystals, which enables its efficiency as a standard drug of choice for multiple comparison studies (Kumar et al., 2016). The early warning signs for the pathogenesis of kidney diseases mainly include the alterations in food and water consumption (Bouanani et al., 2010, Hunter et al., 2014). The RBW monitoring among animal groups up to 3-week postulated that EG and AC-treated group selectively showed aversion towards food as the RBW relatively decrease in 2 and 3-week simultaneously. On the converse, regain in RBW of rats was find out in 6-shogaol and Cystone treated groups showing significance in further disease progression. Similarly, previous reported data postulated the efficacy of DCRE and Cystone treatment prevented further renal impairment and recurrence rate by modifying the hemodynamic changes that were commonly observed with urolithiasis (Drake, P., 2015, Aggarwal et al., 2014, Zhang et al., 2012). In a previous study, EC-treated group link to the formation of various polymorphic CaOx crystals with the different structural habits in collected urine samples for analysis (Doddola et al., 2008). On the contrary, animal groups that received DCRE treatment. These findings are evidence that the antiurolithic activity of DCRE assigns to the presence of polyphenolic compounds in extracts that was confirmed further by employing precision-based analytical evaluation through HPLC fingerprinting (Khan, 1997).

The various molecular mechanism through which 6-Shogaol protects against renal IR injury is by attenuating the canonical NF-κB pathway and activation of heme oxygenase (HO)-1 synthesis via p38 MAPK activation that may correlate clinical evident and efficacy of 6 shogaol in urolithiasis animal models (Han et al., 2019).

## 5 Conclusion

The present study attributed clinical shreds of evidence first time that claiming the significant antiurolithic property of 6-shogaol and its ability to ameliorate different biochemical parameters alterations caused due to calcium oxalate crystal deposition. Further evaluation of intracellular enzymes suggested that treatment with 6 shogoal inhibit calcium oxalate crystal induced renal injuries and protects the kidneys. Hence the current experimental outcomes inferred that administration of 6 shogaol can prevent the urolithiasis and protect the kidneys via inhibition of injuries caused by the crystal deposition and neutralization of reactive oxygen species. This may prove to develop cost-effective alternatives for the cure of urolithiasis.

## Declaration of Competing Interest

The authors declare that there are no conflicts of interest.

## References

[b0005] Aggarwal D., Kaushal R., Kaur T., Bijarnia R.K., Puri S., Singla S.K. (2014). The most potent antilithiatic agent ameliorating renal dysfunction and oxidative stress from Bergenia ligulata rhizome. J. Ethnopharmacol..

[b0010] Alatab, S., Pourmand, G., El Howairis, M. E. F., Buchholz, N., Najafi, I., Pourmand, M. R., Mashhadi, R., Pourmand, N., 2016. National profiles of urinary calculi (a comparison between developing and developed worlds). 26921745

[b0015] Albayrak A., Bayir Y., Halici Z., Karakus E., Oral A., Keles M.S., Colak S., Zipak T., Dorman E., Uludag K. (2013). The biochemical and histopathological investigation of amlodipine in ethylene glycol-induced urolithiasis rat model. Ren. Fail..

[b0020] Anand R., Patnaik G., Kulshreshtha D., Dhawan B. (1994). Activity of certain fractions of Tribulus terrestris fruits against experimentally induced urolithiasis in rats. Indian J. Exp. Biol..

[b0025] Bano H., Jahan N., Makbul S.A.A., Kumar B., Husain S., Sayed A. (2018). Effect of Piper cubeba L. fruit on ethylene glycol and ammonium chloride induced urolithiasis in male Sprague Dawley rats. Integrative Med. Res..

[b0030] Basavaraj D.R., Biyani C.S., Browning A.J., Cartledge J.J. (2007). The role of urinary kidney stone inhibitors and promoters in the pathogenesis of calcium containing renal stones. EAU-EBU Update Series.

[b0035] Baumann J.M. (1998). Stone prevention: why so little progress?. Urol. Res..

[b0040] Bouanani S., Henchiri C., Migianu-Griffoni E., Aouf N., Lecouvey M. (2010). Pharmacological and toxicological effects of Paronychia argentea in experimental calcium oxalate nephrolithiasis in rats. J. Ethnopharmacol..

[b0045] Bouatia M., Benramdane L., Idrissi M.O.B., Draoui M. (2015). An epidemiological study on the composition of urinary stones in Morocco in relation to age and sex. African J. Urol..

[b0050] Chen H., Fu J., Chen H., Hu Y., Soroka D.N., Prigge J.R., Schmidt E.E., Yan F., Major M.B., Chen X. (2014). Ginger compound [6]-shogaol and its cysteine-conjugated metabolite (M2) activate Nrf2 in colon epithelial cells in vitro and in vivo. Chem. Res. Toxicol..

[b0055] Doddola S., Pasupulati H., Koganti B., Prasad K.V. (2008). Evaluation of Sesbania grandiflora for antiurolithiatic and antioxidant properties. J. Nat. Med..

[b0060] Drake, P., 2015. Species Differences in Urinary Specific Gravity of Various Nonhuman Primates.

[b0065] Dugasani S., Pichika M.R., Nadarajah V.D., Balijepalli M.K., Tandra S., Korlakunta J.N. (2010). Comparative antioxidant and anti-inflammatory effects of [6]-gingerol,[8]-gingerol,[10]-gingerol and [6]-shogaol. J. Ethnopharmacol..

[b0070] Evan A.P. (2010). Physiopathology and etiology of stone formation in the kidney and the urinary tract. Pediatric Nephrol..

[b0075] Han S.J., Kim M., D’agati V.D., Lee H.T. (2019). 6-Shogaol protects against ischemic acute kidney injury by modulating NF-κB and heme oxygenase-1 pathways. Am. J. Physiol.-Renal Physiol..

[b0080] Huang H.-S., Ma M.-C., Chen J., Chen C.-F. (2002). Changes in the oxidant-antioxidant balance in the kidney of rats with nephrolithiasis induced by ethylene glycol. J. Urol..

[b0085] Hunter J., Butterworth J., Perkins N., Bateson M., Richardson C. (2014). Using body temperature, food and water consumption as biomarkers of disease progression in mice with E μ-myc lymphoma. Br. J. Cancer.

[b0090] Karadi R.V., Gadge N.B., Alagawadi K., Savadi R.V. (2006). Effect of Moringa oleifera Lam. root-wood on ethylene glycol induced urolithiasis in rats. J. Ethnopharmacol..

[b0095] Khan S. (1997). Animal models of kidney stone formation: an analysis. World J. Urol..

[b0100] Khan S.R. (2013). Reactive oxygen species as the molecular modulators of calcium oxalate kidney stone formation: evidence from clinical and experimental investigations. J. Urol..

[b0105] Knoll T., Schubert A.B., Fahlenkamp D., Leusmann D.B., Wendt-Nordahl G., Schubert G. (2011). Urolithiasis through the ages: data on more than 200,000 urinary stone analyses. J. Urol..

[b0110] Kou X., Wang X., Ji R., Liu L., Qiao Y., Lou Z., Ma C., Li S., Wang H., Ho C.-T. (2018). Occurrence, biological activity and metabolism of 6-shogaol. Food Funct..

[b0115] Kumar B., Wadud A., Jahan N., Sofi G., Bano H., Makbul S.A.A., Husain S. (2016). Antilithiatic effect of Peucedanum grande CB Clarke in chemically induced urolithiasis in rats. J. Ethnopharmacol..

[b0120] Kvsrg P., Sujatha D., Bharathi K. (2007). Herbal drugs in urolithiasis-a review. Pharmacog Rev.

[b0125] Lulat S.I., Yadav Y.C., Balaraman R., Maheshwari R. (2016). Antiurolithiatic effect of lithocare against ethylene glycol-induced urolithiasis in Wistar rats. Indian J. Pharmacol..

[b0130] Neisius A., Preminger G.M. (2013). Epidemiology, prevention and redefining therapeutic standards. Nat. Rev. Urol..

[b0135] Prasad K., Bharathi K., Srinivasan K. (1993). Evaluation of Musa (Paradisiaca Linn. cultivar)-“Puttubale” stem juice for antilithiatic activity in albino rats. Indian J. Physiol. Pharmacol..

[b0140] Rao M., Rao M. (1998). Protective effects of cystone, a polyherbal ayurvedic preparation, on cisplatin-induced renal toxicity in rats. J. Ethnopharmacol..

[b0145] Sedeek M., Nasrallah R., Touyz R.M., Hébert R.L. (2013). NADPH oxidases, reactive oxygen species, and the kidney: friend and foe. J. Am. Soc. Nephrol..

[b0150] Selvam R., Kalaiselvi P., Govindaraj A., Murugan V.B., Kumar A.S. (2001). Effect of A. lanata leaf extract and Vediuppu chunnam on the urinary risk factors of calcium oxalate urolithiasis during experimental hyperoxaluria. Pharmacol. Res..

[b0155] Semwal R.B., Semwal D.K., Combrinck S., Viljoen A.M. (2015). Gingerols and shogaols: Important nutraceutical principles from ginger. Phytochemistry.

[b0160] Seow S.L.S., Hong S.L., Lee G.S., Abd Malek S.N., Sabaratnam V. (2017). 6-shogaol, a neuroactive compound of ginger (jahe gajah) induced neuritogenic activity via NGF responsive pathways in PC-12 cells. BMC Complement. Alternative Med..

[b0165] Tiselius H. (2003). Epidemiologie a medikamentózní léčba urolitiázy. BJU Int..

[b0170] Trinchieri A. (2008). Epidemiology of urolithiasis: an update. Clin. Cases Mineral Bone Metabolism.

[b0175] Tugcu V., Kemahli E., Ozbek E., Arinci Y.V., Uhri M., Erturkuner P., Metin G., Seckin I., Karaca C., Ipekoglu N. (2008). Protective effect of a potent antioxidant, pomegranate juice, in the kidney of rats with nephrolithiasis induced by ethylene glycol. J. Endourol..

[b0180] Yi J.-K., Ryoo Z.-Y., Ha J.-J., Oh D.-Y., Kim M.-O., Kim S.-H. (2019). Beneficial effects of 6-shogaol on hyperglycemia, islet morphology and apoptosis in some tissues of streptozotocin-induced diabetic mice. Diabetol. Metabolic Syndrome.

[b0185] Zhang C.-Y., Wu W.-H., Wang J., Lan M.-B. (2012). Antioxidant properties of polysaccharide from the brown seaweed Sargassum graminifolium (Turn.), and its effects on calcium oxalate crystallization. Mar. Drugs.

[b0190] Zhang J., Wang Q., Xu C., Lu Y., Hu H., Qin B., Wang Y., He D., Li C., Yu X. (2017). MitoTEMPO prevents oxalate induced injury in NRK-52E cells via inhibiting mitochondrial dysfunction and modulating oxidative stress. Oxidative Med. Cell. Longevity.

